# The Middle Lamella of Plant Fibers Used as Composite Reinforcement: Investigation by Atomic Force Microscopy

**DOI:** 10.3390/molecules25030632

**Published:** 2020-02-01

**Authors:** Alessia Melelli, Olivier Arnould, Johnny Beaugrand, Alain Bourmaud

**Affiliations:** 1 IRDL, Université de Bretagne Sud, UMR CNRS 6027, 56321 Lorient, France; alain.bourmaud@univ-ubs.fr; 2 LMGC, Université de Montpellier, CNRS, 34095 Montpellier, France; olivier.arnould@umontpellier.fr; 3 INRAE, UR1268 BIA Biopolymères Interactions Assemblages, 44316 Nantes, France; johnny.beaugrand@inra.fr

**Keywords:** biocomposite, plant fibers, middle lamella, AFM PF-QNM, nanomechanical characterization

## Abstract

Today, plant fibers are considered as an important new renewable resource that can compete with some synthetic fibers, such as glass, in fiber-reinforced composites. In previous works, it was noted that the pectin-enriched middle lamella (ML) is a weak point in the fiber bundles for plant fiber-reinforced composites. ML is strongly bonded to the primary walls of the cells to form a complex layer called the compound middle lamella (CML). In a composite, cracks preferentially propagate along and through this layer when a mechanical loading is applied. In this work, middle lamellae of several plant fibers of different origin (flax, hemp, jute, kenaf, nettle, and date palm leaf sheath), among the most used for composite reinforcement, are investigated by atomic force microscopy (AFM). The peak-force quantitative nanomechanical property mapping (PF-QNM) mode is used in order to estimate the indentation modulus of this layer. AFM PF-QNM confirmed its potential and suitability to mechanically characterize and compare the stiffness of small areas at the micro and nanoscale level, such as plant cell walls and middle lamellae. Our results suggest that the mean indentation modulus of ML is in the range from 6 GPa (date palm leaf sheath) to 16 GPa (hemp), depending on the plant considered. Moreover, local cell-wall layer architectures were finely evidenced and described.

## 1. Introduction

In the last few decades, plant fiber-reinforced composites were progressively developed to replace composites where synthetic fibers are usually used or, in some cases, to create new families of composites having specific properties [1]. Their cost-effective production, low environmental impact, and specific mechanical properties, almost comparable to those of glass fibers, encourage industries to invest in this area [2,3].

Many plant species in nature produce fibers that can be employed for this purpose, but their structure, chemical composition, and properties differ greatly and depend on the type of plant [4,5,6]. An exhaustive summary of the plant fibers used for composite materials can be found in Reference [7], where they are divided into wood and non-wood (e.g., bast), based on their location inside the plant. Bast fibers have high cellulose content, low microfibrillar angle (MFA), and consequently high mechanical performances in the fiber axis (or longitudinal) direction. Thus, they play a central role in new biocomposites, especially compared to leaf, xylem, or mesocarp fibers, and a clear example can be found in Wambua et al. [8]. The authors studied several poly-(propylene) composites reinforced with different plant fibers (sisal, kenaf, jute, hemp, and coir), and their results showed that coir fibers, extracted from seeds, have lower longitudinal mechanical properties than the others but, on the other hand, they exhibit a higher impact strength, which was also confirmed in Reference [9]. This result follows the functional evolution of the different kinds of cells in a plant; for example, bast fibers are responsible for the stiff structure of a plant and this specific role also explains their high mechanical performance. 

**Figure 1:**
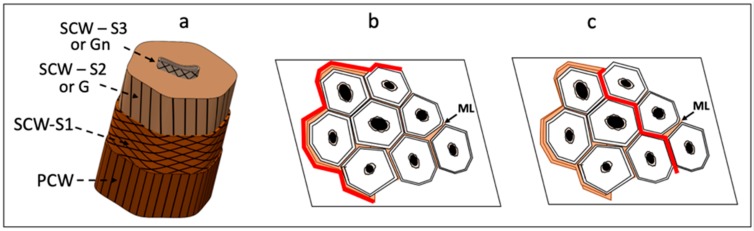
Diagrams illustrating the structural arrangement of a single flax fiber (**a**) and a crack (in red) that (**b**) propagates along the perimeter of a bundle and (**c**) through the bundle in a plant fiber-reinforced thermoset composite under tensile tests. SCW S_1_, S_2_ or G, S_3_ or Gn = secondary cell wall, PCW = primary cell wall, ML = middle lamella.

Every bast fiber has a similar (ultra)structural model even if they originate from different plants. An elementary fiber is a single cell, and several fibers are linked to each other to form a bundle of several dozens of single fibers having a multilayer structure, as illustrated in Figure 1a: (1) the lumen is the central hollow part of the cell and its shape and diameter vary with the maturity of the plant and the environmental conditions during growth; (2) the secondary cell wall is the thickest layer, divided into two to three sub-layers (S_1_, S_2_ or G, and S_3_) rich in cellulose where the thinner and not always visible S_3_ can also be assimilated to unmatured Gn layer instead of a real S_3_ [10]; (3) the primary cell wall is the external layer enriched in hemicelluloses, pectins, and lignin [11]. Between the fibers, there is another layer called middle lamella (ML), which cements the primary cell walls of adjacent cells together and is mainly composed of pectic polysaccharides, lignin, and a small amount of proteins [12,13,14]. This binder layer between two cells acts as a very thin and efficient interfacial matrix in the plant [13,15]. 

Despite this complex hierarchical structure, the S_2_ is the most important layer and the main contributor to the longitudinal mechanical properties of a single fiber due to its thickness and ultra-structure. Consequently, it is also the layer most responsible for the mechanical behavior of a final product when plant fibers are used as reinforcements in composite materials. The conventional interface level between polymer matrix and plant fibers, which often coincides with the secondary wall, was deeply investigated. However, middle lamellae have a strong presence in plant fiber composite materials due to the specific arrangement of the fibers into bundles in planta. Their amount depends not only on retting and extraction conditions but also on the material processing conditions that influence the shear rate and the temperature experienced by the fibers during composite processing [16,17,18]. 

Some studies demonstrated how this layer plays a central role in the mechanical behavior of the final product. Bourmaud et al. [19] noted several failure mechanisms for plant fiber biocomposite materials, and Monti et al. [20] showed that, in thermoset composites, cracks often propagate around the fiber bundles and through them, as illustrated in Figure 1. Recently, in Reference [21] results showed that middle lamellae constitute an area of failure and that their behavior is strongly related to their properties and cohesion with the fibers. 

For a correct computational modeling of the plant fibers, the plant fiber bundles, and the biocomposite mechanical behavior, numerical values of mechanical properties of the middle lamella are required [22]. Some attempts were made to measure the mechanical properties of the ML of onion or flax cells using tensile tests [23,24], but this kind of test remains challenging and difficult to interpret. Due to its reduced thickness in the order of the sub-micron, most of the measurements were done using micromechanical characterization techniques, such as nanoindentation in the cell corner middle lamella (CCML); however, to our best knowledge, they are mainly limited to some wood samples [25,26,27,28]. In these cases, the average indentation modulus obtained ranges from around 5 to 11 GPa but with a generally high standard deviation.

On the other hand, atomic force microscopy (AFM) was already used to investigate the middle lamellae [28]. However, even if this technique is suitable for performing mechanical measurements in areas at the nanoscale level like the CML layer, to our best knowledge, available data are scarce and limited to the middle lamella of wood using contact resonance-AFM (CR-AFM) [29,30] or AFM peak-force quantitative nanomechanical property mapping (PF-QNM) [31] with an average value ranging from 6 to 17 GPa, and of bamboo, using AFM PF-QNM [32], with an average value around 14 GPa [31]. These two kinds of measurements, i.e., nanoindentation and almost all the mechanical AFM methods, give access to the indentation modulus that depends on all the elastic constants of the material (Young’s moduli, shear moduli, and Poisson’s ratios) in a complex manner for an anisotropic behavior like the secondary cell-wall layers [33,34]. However, in this case, as there are no microfibrils in the middle lamella and its structure is quite random, the ML is usually considered to have an isotropic elastic behavior [35] and the indentation modulus only depends on one Young’s modulus and one Poisson’s ratio.

Until now, few experimental data on its independent elastic properties are available, and only an estimate of the elastic properties of each component extracted from the middle lamella is known. On the contrary, their biochemical composition is quite well documented in the literature. To our best knowledge, only Zamil et al. [23] tried to measure its tensile properties in onion, and they obtained mean values of the Young’s modulus ranging from around 8 to 11 GPa. In the case of an isotropic material, the indentation modulus *M* can be expressed according to the Hertz formula, *M* = *E*/(1 − *ν* ^2^), where *E* is the Young’s modulus and ν is the Poisson’s ratio. The Poisson’s ratio of the middle lamella is not known but it should be lower than 0.5 like most amorphous polymer blends. Thus, in this case, the indentation modulus is quite close to the Young’s modulus. 

In previous papers, a protocol to study the mechanical properties of plant cells with the AFM PF-QNM method was already tested and the results were validated [36,37,38,39]. In the present work, AFM PF-QNM was used to investigate the area of the middle lamellae at the cell-wall level and to obtain information on their indentation modulus. For that purpose, a panel of plant fibers commonly used in biocomposites, i.e., flax, hemp, jute, kenaf, nettle, and date palm leaf sheath, was investigated. A database of middle lamella indentation modulus was addressed on this range of plant fibers to implement the knowledge of this layer and help future works in computational modeling. 

## 2. Results and Discussion

**Figure 2:**
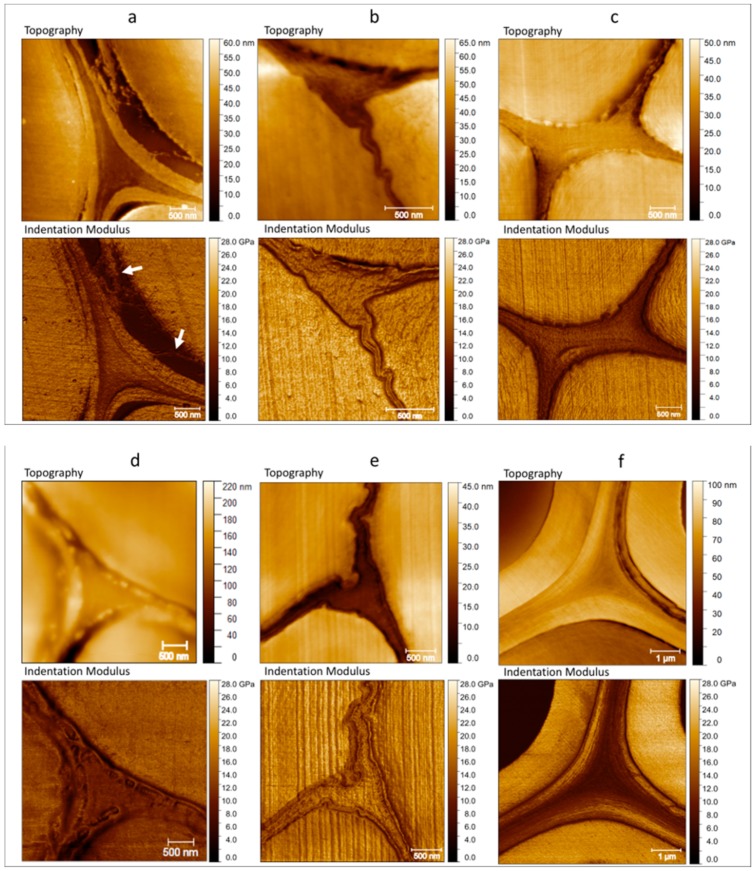
Topography (top) and indentation modulus (bottom) of the cell corners of (**a**) flax with illustration of decohesion (highlighted with white arrows) between S_1_ and S_2_ layers, (**b**) nettle with values of indentation modulus close to those of the cell wall, (**c**) jute, (**d**) kenaf, (**e**) hemp with the highest indentation modulus, and (**f**) date palm tree with the lowest indentation modulus but with a homogeneous and thick area for the middle lamella.

Figure 2 shows the topography and indentation modulus mapping obtained through AFM PF-QNM measurement of flax, nettle, jute, kenaf, hemp, and date palm fibers. These images not only provide values of the indentation modulus but also information about the morphology of the middle lamellae. In References [40,41], the authors made a distinction between the middle lamella (ML) and the compound middle lamella (CML), especially referring to the wood samples, but this distinction is also applicable to other plant fibers [13]. In fact, the middle lamella between two primary walls of two different cells is often not distinguishable [13], and the small tripartite layer appears as a single layer, such as in Figure 2b or Figure 2d. For this reason, in the present paper, the indentation modulus is measured in the tricellular junctions, usually called the cell corner middle lamella (CCML), where the middle lamella is well discernible. However, it should be taken into account that the biochemical composition of the CCML may differ from the ML [13].

In Figure 2a, flax shows an interesting fracture (decohesion highlighted with white arrows), which probably occurred between the secondary S_2_ and S_1_ cell wall layers during the handling of the green flax stem. In several other images of the same section of the flax stem, other cells showed this kind of fracture between S_1_ and S_2_ secondary layers, which was previously noted by Arnould et al. [39] and Goudenhooft et al. [38]. In addition, from Day et al.’s (2005) [42] pictures, it is possible to identify fractures, which occurred at the CML-S_1_ or S_1_-S_2_ level and their observations are in line with our results and conclusions. Le Duigou et al. [43] also observed peeling failures of the external fiber layer of flax fibers during a microdroplet debonding test, proving the low cohesion degree between structural layers.

In addition, the images collected confirm the observations done by Zamil and Geitmann [13] and Raghavan et al [15] in the ML and S_1_–S_2_ region. The authors reported that the middle lamella is stronger than the primary or secondary wall layers and not damaged from a mechanical stretch; on the contrary, fractures take place in the adjacent primary or secondary cell-wall layers. This fact reinforces the generally accepted assumption in the plant fiber community that the mechanical behavior of the ML is far from the brittle behavior associated with the cell-wall layers. Middle lamellae in flax are well distinguishable and with an indentation modulus clearly lower than the other cell-wall layers, as can be expected given that this layer has a random organization and constitutes non-cellulosic polymers. For the same variety of flax investigated by our team [36,38], a similar contrast was highlighted between values of indentation modulus obtained in S_2_ and ML, even if measurements were done at a larger scale and not especially dedicated to the investigation of the ML. Possible differences in thickness or arrangement of the ML area can be due to the position of the fibers inside the same plant. In fact, one can find changes in the lumen, the cell, and the size of the middle lamella according to the considered stem cross-section along the height of the stem [44]. 

Nettle is reported in Figure 2b, and its middle lamella clearly shows a high indentation modulus, almost comparable to the surrounding cell walls. In general, the indentation modulus is highly influenced by the anisotropic character of the considered material; thus, in this case, of quasi isotropic non-cellulosic polymer (NCP) middle lamellae, the indentation modulus can be in the same range as the S_2_, which is underestimated due to the highly anisotropic nature of the secondary cell wall [33]. However, the indentation modulus in the middle lamella is remarkably high here and could be linked to a specific biochemical composition that must be investigated. 

**Figure A1:**
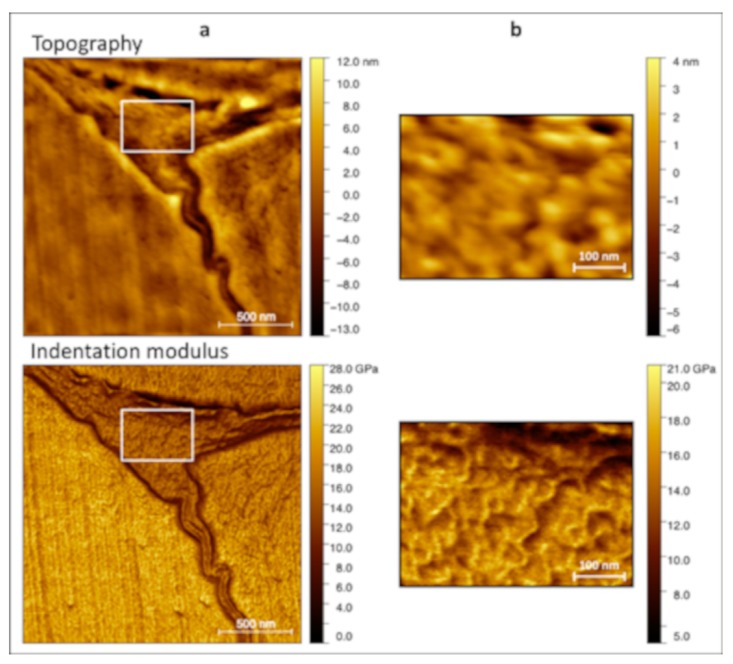
(**a**) Surface topography and indentation modulus of nettle corresponding to those reported in Figure 2. (**b**) Zoomed-in view of the ML region, corresponding to the white rectangle in (**a**), in order to highlight the correlation between topography and indentation modulus.

The indentation modulus in the middle lamella is globally homogeneous, but one can note small areas where the indentation modulus is lower and other areas where it is higher, creating an irregular matrix like a grid or globular aspect. Considering the size of these nodules, of a few tens of nanometers (Figure A1, Appendix A, where zoomed-in view of the ML region are shown, not corrected for the effect of tip dilation), they might be attributed to the lignin [31,45,46], but the variations of the modulus measured in that area are more likely due to a topographic effect. As the roughness in that area is similar to the tip radius, it induces a strong correlation between the topography and the indentation modulus (Figure A1, Appendix A), which induces an apparently higher indentation modulus between two nodules and a lower one on top of them [47]. Bourmaud et al. [36] hypothesized that the degree of lignification is an important factor in the middle lamella morphology; however, although it is known in the literature that this layer is enriched in lignin, a direct quantification remains a challenge because of its sub-micrometric scale. In addition, in nettle, as previously mentioned, the primary cell wall is not well distinguishable from the middle lamellae, and they appear as a single unitary layer between two fibers. 

**Figure 3:**
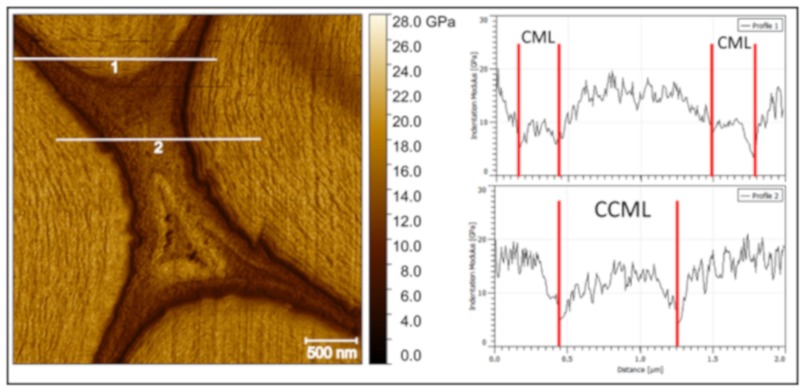
Middle lamella cell corner in jute fibers. Two profiles (in white) are marked on the indentation modulus mapping, and graphics extracted are shown on the right. The middle lamella cell corner (CCML) shows an increment of the values of indentation modulus from the edge, near the fiber cells, to the core.

On the other hand, the middle lamellae of jute fibers are clearly distinguishable from the surrounding cell walls (Figure 2c) thanks to their lower indentation modulus. In addition, the indentation modulus in CCML appears homogeneous with a fine texture that does not represent disconnections or asperities when compared with those of nettle. However, one can note that, in jute, there is a small difference between the indentation modulus of the central area of the CCML and its edge (Figure 3) that is not due to an effect of topography. The indentation modulus gradually increases from the edge to the center of the tricellular junction; this singular phenomenon was noted on each jute CCML investigated but not for other middle lamellae investigated in the present paper, such as nettle and palm sheath fibers. The AFM PF-QNM method, thanks to its high resolution, is able to clearly highlight this kind of gradient in a restricted area (Figure 3), confirming its potential for ultra-local mechanical investigations. 

In Figure 2d, the primary walls of kenaf fibers show strong inhomogeneities with a highly globular aspect. In the present case, even more than in the case of nettle, the topography of the nodules induces a strong effect on the apparent indentation modulus. Moreover, the distinction of the middle lamella and the primary walls is difficult compared to other plant fibers. The origin of these nodules is not clear but might be linked to the cutting behavior and, therefore, to the mechanical properties of the different components of the cell wall layers during the sample surface preparation. Softer components make them harder to cut and leads to more irreversible deformation when cutting. These differences in behavior induce variations in topography [29,48] such as steps between layers of the cell wall (as between S_1_ and S_2_ or between S_2_ and the embedding resin) and roughness or nodules, as in the case of lignin, within a layer. However, biochemical investigations, like Raman spectroscopy, must be done to better understand this phenomenon.

Figure 2e illustrates a tricellular junction between three hemp fibers. The middle lamella of hemp, together with the nettle, shows the highest values of indentation modulus that are also well correlated with values found by Bourmaud et al. [36] on several plant fibers studied by AFM PF-QNM mode. Nevertheless, in both nettle and hemp, not only the middle lamella but also the indentation modulus of hemp and nettle fiber cell walls are high. The matrix inside the middle lamella of hemp is similar to the grid texture that was observed in nettle; however, in addition to this morphology and contrarily to the nettle, the primary cell wall is clearly distinguishable from the ML. 

**Figure 4:**
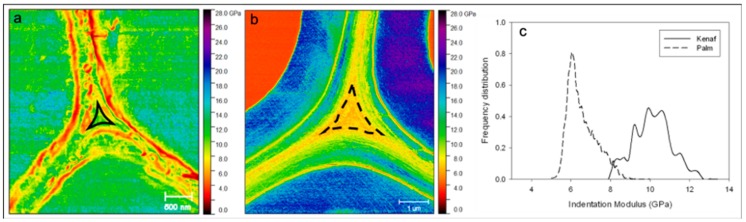
(**a**) Kenaf middle lamella where a non-uniform distribution is clearly visible; (**b**) date palm middle lamella with a more uniform indentation modulus distribution inside the middle lamella of the tricellular junction; (**c**) distribution of indentation modulus in middle lamella area of kenaf and palm, showing a high difference in the spread of values. For kenaf and date palm, the area investigated is highlighted in (**a**) and (**b**) using solid and dotted black lines, respectively.

In Figure 2f, date palm CCML is demonstrated to have the lowest values and an extreme homogeneity of the indentation modulus with a clear separation of the layers from each other. This is especially visible in Figure 4 where the middle lamellae of kenaf and date palm fibers are compared and underlined. Both graphical representations and distributions of the indentation modulus (Figure 4c) underline significant differences in terms of indentation modulus between the two plants considered.

In kenaf, the non-uniformity of color mapping clearly shows discontinuities with higher and lower values of the indentation modulus. These heterogeneities are partly due to a topography effect of the mechanical measurements caused by the huge globular aspect of the primary wall and possibly due to the cutting effects during the sample preparation between cell-wall layers or within a layer. On the other hand, the indentation modulus in the date palm middle lamella is more uniform, although the surface area analyzed is larger than that for kenaf.

**Figure 5:**
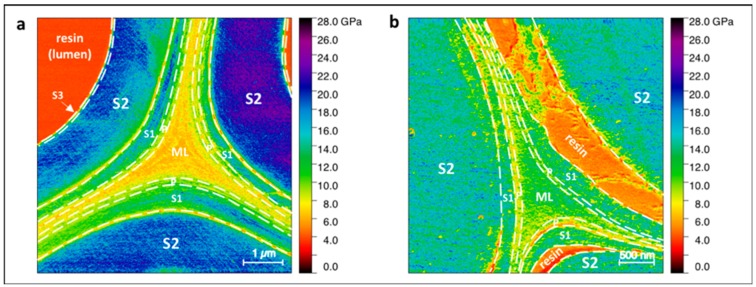
(**a**) Date palm leaf sheath and (**b**) flax middle lamella area; limits between layers are clearly identified and highlighted by dotted lines.

For some fibers such as flax and palm, it is possible to trace and identify all the layers present in the fiber bundles, which are underlined thanks to their different modulus in Figure 5, highlighting the typical hierarchical structure. 

**Figure 6:**
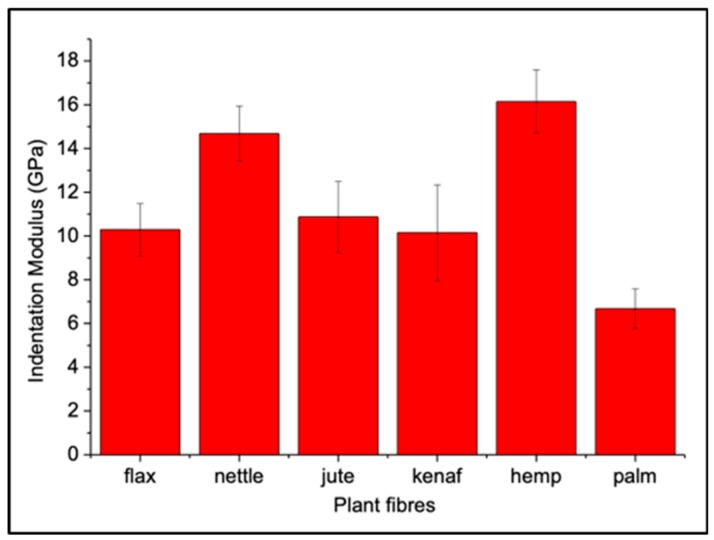
Average values of indentation modulus calculated in each plant fiber middle lamella considered and respective standard deviation bars.

Average numerical values of the indentation modulus calculated in a selected area of each middle lamella are summarized in Figure 6 and Table 1 with their respective standard deviation. 

As previously mentioned, kenaf has the highest standard deviation since these measurements reflect the important inhomogeneity (see Figure 4a) of the CCML region. Nevertheless, average values from flax, jute, and kenaf are comparable. Hemp and nettle have the highest indentation modulus for the middle lamellae. The morphology of these two ML is similar, probably because of the presence of a large amount of lignin. On the other hand, palm fiber CCML regions show the lowest indentation modulus.

The different origin of the fiber elements is also visible in their overall chemical composition (Table 2), which is related to their botanical functions or roles for the plant. Among the six plant species considered, some fibers ensure the role of conduction of raw or elaborated sap, and others have the function of supporting the stem and ensure its stability. For example, kenaf fibers are located in the bast (cortical layer) and core (woody) region. The bast fibers constitute around 40% of the total amount of the fibers. Primary phloic fibers (PPF) from procambium in the protophloem region and secondary phloic fibers (SPF) from cambium are both developed in both jute [49] and kenaf. Thus, in these two plants, the chemical composition of the fibers is highly dependent on the tissue from which they come. This kind of cellular heterogeneity, coming from the tissue origin, does not exist for flax and nettle, which have gelatinous and poorly lignified fibers that can be extracted only from the primary phloem region. In the case of hemp, a possible mix between primary and secondary fibers may occur; these two kinds of fibers have a role in the mechanical support of the plant. Consequently, their compositions and mechanical properties are fairly similar and the main difference is the length, which is significantly shorter for the secondary fibers because of their delayed growth that occurs after the structuration of the tissues. 

For flax, hemp, and jute, middle lamellae are often reported as lignin-enriched domains, which are often highlighted to explain the mechanical stiffness and cohesion of the fiber bundles employed as composite reinforcement. Nevertheless, cell-wall lignification generally appears after seed maturity and, according to the cultivation management, can be strongly limited. For flax pulled out after flowering or seed maturity, Day et al. [42] reported a low lignin labeling in the CML area, absent in the cell corner and tricellular junctions, regardless of the epitope considered. Flax samples investigated in our paper come from the plant stem and they were collected before the lignification of the middle lamella; in addition, no retting or scutching processes were performed contrary to the hemp fibers. In this case, the lignification and the retting effect could explain the higher indentation modulus of hemp fibers than of flax. 

As previously reported, the amount of lignin and the quality of links between the several layers influence the behavior of the fiber bundles and, in the specific case of flax where decohesion between CML-S_1_ or S_1_–S_2_ level is noted, our results are in line with the figure reported in Reference [42]. These damages at the level of the middle lamella also have an impact on the biocomposite material when flax fibers are used as reinforcement. A second polymer well known to be an influent actor of the cell cohesion is the pectin family, precisely located in the ML. It was demonstrated that the mechanical properties of the ML may vary with the degree of the esterified form of homogalacturonan [50]. This pectin is then more or less able to generate interchain interactions, for instance, by calcium cross-linking, if homogalacturonans are de-esterified. From our panel of phenotype samples, the backbone of the pectin methylation degree is arguably different, and some contrasted mechanical properties can be, therefore, expected due to susceptibility to cross-linking. 

Dhakal et al. [37] investigated the damage mechanisms after impact testing for palm/PCL composites and they reported that, even after testing, the morphology of the date palm bundles remained unaltered. This is due to the high content in lignin and the homogeneity of the middle lamella (Figure 2f) that strongly links the single palm fibers together, more than in other plant fibers, despite a soft middle lamella (Figure 6 & Table 1). The silica-reinforced external paravascular or parafibrovascular parenchyma of bundles [51] is arguably too strong to be damaged, even after a high shear-rate process. In Reference [52], opposite results were highlighted on flax bundles and significant cracks were visible after transverse tensile tests on the CML area. Indeed, the individualization of the fibers can be more or less pronounced, according to the process used to produce the composite material [18] and the scutching or hackling degree of the fibers [53] during the fiber extraction process. Consequently, all these factors can introduce significant changes in the mechanical performance of the final composite. In addition, when the composite contains a large number of flax fiber bundles, one can notice that, during loading, the damages occur preferably in the bundles, especially in the CML area [19,20]. In this case, the fiber–matrix interface is not the most critical, even when the polyolefin matrix, with a weak fiber–matrix interface, is used. Thus, even if the indentation modulus of flax ML is higher than the palm one (Table 1), the individualization of flax fibers is facilitated thanks to the specific organization of the bundles and preliminary action of retting and scutching. However, as reported in Reference [13], ML in flax are not directly involved in the failures, but the weak area seems to be between the different layers (S_1_–S_2_) and not inside the middle lamella, even if ML exhibits a lower stiffness. A hypothesis for this result is that the softness and the cohesion of the ML are probably an advantage for the energy dissipation, and they limit the cell-wall damage during mechanical loading or shear rate stress. To support that assumption, a parallel can be done with the nacre, where the microstructure displays some homologies with the lignocellulosic bundles. Indeed, stiff and large structures of ceramic, as the fiber cell wall in our study, are embedded in a thin and weak mortar made of unfolded proteins and chitin biopolymers. The latter is the middle lamella of the bundles. During loading, in the nacre, the thin interface is able to deform, with most of the fracture occurring at the interfaces, providing more inelastic regions and favoring more energy dissipation and higher work of fracture, thereby giving nacre an elevated damage tolerance [54].

In our plant samples, palm sheath fibers, which have CCML with a lower indentation modulus, are probably less damaged in contrast to the flax fibers. 

On the other hand, if we consider the jute fibers, it is clear that they have an indentation modulus similar to flax as reported in this paper (see Table 1), but their mechanical behavior inside a composite material is very different. This is probably due to their high lignin content, as well as to their short length, and, as a consequence, high volume content of middle lamellae. The cohesion in bundles is strong, inducing a specific behavior and a flow orientation in extrusion or injection molding [55]. Moreover, a high lignification is in favor of a good fiber–matrix interface, and Graupner et al. [56] noted that lignin may be an interesting adhesion promoter. Thus, flax, jute, and kenaf have similar CML indentation modulus (Table 1), but their mechanical and morphological behavior differs widely.

Finally, other fibers, such as hemp, have a strong tendency to be divided or fibrillated during the composite production process. This phenomenon was highlighted in extruded or injected compounds [57,58,59]. Interestingly, the difference between CML and S_2_ modulus is lower for hemp compared to other plants, and this might introduce a strong link between these two layers, whereby a weaker interface could possibly be identified at the intra S_2_ layer level. 

Nevertheless, it is important to keep in mind that the morphology of the fibers may be involved in the breaking mechanism, and that the length of fibers and the ratio between the cell wall and lumen area may affect the critical shear rate, leading to irreversible damage. In addition, the indentation modulus is not necessarily correlated to the structural cohesion between layers, and the hardness investigated by the nano-indentation technique can be a better indicator even though it has a lower resolution than AFM PF-QNM. It is interesting to note that Wimmer et al. [28] studied the relationship between the longitudinal indentation modulus and hardness of CCML and S_2_ in spruce wood, and they found a strong relationship between the indentation modulus of the CCML with its hardness, i.e., stiffer denoting harder, unlike the S_2_. In this article, the indentation modulus of the CCML ranges from 4 to 12 GPa, whereas the hardness ranges from 0.12 to 0.47 GPa with a mean value close to that of the S_2_. As the hardness depends on the inelastic behavior of a material, it is linked to its plastic behavior and property at break. If we assume that these results can be transposed here, it would mean that nettle and hemp fiber middle lamellae have a higher hardness in relation to a higher secondary wall longitudinal property, contrary to palm leaf sheath fibers. This seems to make sense mechanically, but needs to be confirmed by additional hardness measurements in our samples and mechanical strength tests of ML [23] or bioinspired films [31].

For this reason, our measurements revealed huge differences in the indentation modulus of the middle lamella according to the plants considered, but it is difficult to correlate them directly to the differences in their mechanical behavior or damage mechanisms when fibers are employed in biocomposites. In fact, these complex phenomena are impacted not only by the nature and cohesion of this middle lamella, but also by the morphology and the biochemical composition or structure of the fibers and bundles.

## 3. Materials and Methods 

### 3.1. Materials

**Figure 7:**
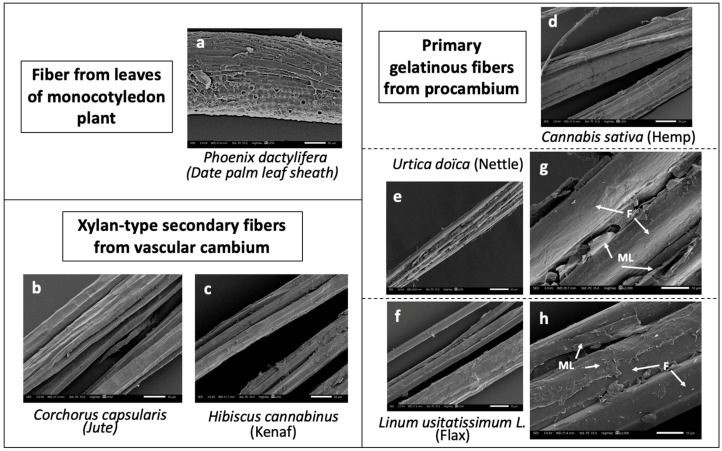
SEM images and classification of date palm leaf sheath (**a**), jute (**b**), kenaf (**c**), hemp (**d**), nettle (**e**), flax (**f**) fibers studied in this work. (**g**) and (**h**) are zooms on middle lamellae area of nettle and flax, respectively (F = fibers, ML = middle lamella). Scale bars correspond to 50-µm lengths for all except (**g**) and (**h**) (10 µm).

Various plant fibers were taken to represent three of the main groups of fibers used for biocomposite manufacturing as reported in Figure 7. 

For flax (*Linum Usitatissimum L.*), stems of the Eden variety cultivated in the year 2015 (Terre de Lin, Normandy, France) were chosen and cut after the first ramification (120 days) [38]. The nettle (*Urtica Dïoica*) samples were harvested in Lorraine (France) in 2014 and stored at room temperature in darkness; then, fibers were manually extracted. Hemp fibers from plants cultivated in 2016 (*Fedora 17 variety*) in Bar-Sur-Aube (France) and field retted were chosen. Jute (*Corchorus capsularis L*) and kenaf (*Hibiscus cannabinus L*) fibers, provided by Derotex (Wielsbeke, Belgium), were grown in Bangladesh, and retted in water before being mechanically extracted from the stems; both were cultivated in 2015 [36]. Date palm fibers (*Phoenix dactylifera L.*) were from Al-Ahsa (Eastern Province of Saudi Arabia), and the large bundle of the mesh surrounding the date palm tree stems were considered [51].

All samples were dried 2 h in an oven at 60 °C to eliminate the moisture; then, they were dehydrated in a graded series of ethanol and included in London Resin (LR)-white acrylic resin. The final polymerization of the resin was made in the oven at 60 °C overnight. 

Embedded samples were then machined to reduce their cross-section to 1 × 2 mm, and an ultramicrotome (Ultracut R, Leica Microsystems SAS, Nanterre, France) with diamond knives (Histo and Ultra AFM, Diatome, Nidau, Switzerland) was used to cut a series of very thin sections (about 50 nm thick in the last step) at reduced cutting speed (~1 mm/s) to minimize compression and sample deformation during the cutting process. This preparation method resulted in much reduced surface roughness, which made it possible to obtain relevant AFM PF-QNM measurements. 

### 3.2. Methods

A multimode atomic force microscope (Bruker) was equipped with a RTESPA-525 probe (Bruker AFM Probes, Camarillo, CA, USA) for the PF-QNM analysis, and the protocol presented in Reference [39] was used. Firstly, the cantilever spring constant was calculated using the Sader method (https://sadermethod.org/). The range of the cantilever stiffness for all the probes used was between 116 and 200 N/m. The tip radius was then calibrated with a relative method using HOPG (highly oriented pyrolytic graphite, 18 GPa indentation modulus, distributed by Bruker) and Kevlar fibers embedded in an epoxy resin (Epofix, Struers, Champigny sur Marne, France) previously measured by nanoindentation, having an indentation modulus around 20 GPa [39]. The tip radius used was between 15 and 50 nm for all the probes. The maximum of the peak force setpoint used was set to 200 nN, with 2 kHz peak force frequency, 40 kHz Low Pass deflection BandWidth, and 75 nm peak force amplitude. A test to check the parameters was made on HOPG and Kevlar fiber/epoxy resin samples. For all measurements, 3–4 acquisitions were done with a verification of the value of the indentation modulus in the embedding resin of each sample. In all cases, since all the tested materials are stiff (more than a few GPa), the tip radius was quite small, the adhesion force was a few tens of nanoNewtons (lower than half the applied force setpoint), and the maximum indentation depth (e.g., of the order of 1 nm) was small compared to the tip radius, we used a Derjaguin-Muller-Toporov (DMT) model (i.e., Hertz model including the effect of adhesion without any change in the contact behavior) [74] to process the force–distance curve and extract the indentation modulus.

**Figure 8:**
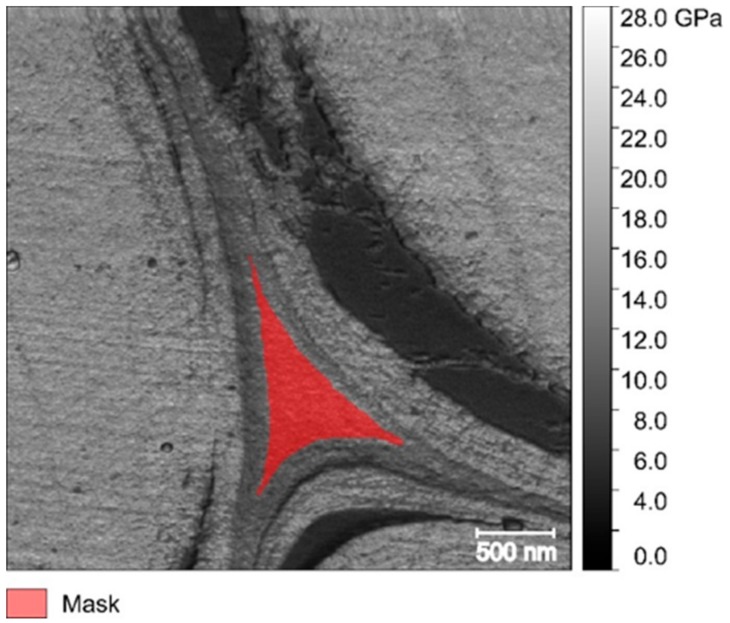
Flax middle lamella with a selected area mask to calculate the indentation modulus mean value and standard deviation.

Gwyddion software (Department of Nanometrology, Czech Metrology Institute, Brno, Czech Republic, http://gwyddion.net [75]) was used to process the images acquired. For each sample, at least three images of 512 × 512 pixels were used and several images of 256 × 256 pixels were acquired and considered to have good image representability and a statistically significant average. A small area of each image was selected according to the morphology of the middle lamella, and only the cell corner (tricellular junctions) was considered, as shown in Figure 8. A mean of the indentation modulus of the selected area was calculated by using the “statistical quantities” tool of the software. 

## 4. Conclusions

The results obtained in this paper show differences in both the elastic mechanical behavior and the morphology of the middle lamellae in fiber bundles from six different plants commonly used as biocomposite reinforcements: flax, hemp, jute, kenaf, nettle, and date palm leaf sheath. Atomic force microscopy mechanical characterization mode (AFM PF-QNM) was used here as it is probably the only technique available today capable of providing semi-quantitative mechanical values of very thin layers at the nanoscale level, such as middle lamellae. This allowed us to propose a comparative database of the indentation modulus of the cell corner middle lamella of the six plant fibers considered with three different groups: the first one (flax, jute and kenaf) with intermediate indentation modulus (on average between 8 and 12 GPa) close to values already obtained in previous studies on wood fiber middle lamellae, the second one (hemp and nettle) with very high values (in average between 13 and 17 GPa), surprisingly close to those of the secondary cell wall, and the last one (date palm leaf sheath) with low values (in average between 6 and 9 GPa), close to the lowest values obtained on wood fiber middle lamella. Moreover, the sample topography highlights morphological differences and heterogeneities between the middle lamella, as well as the primary and the secondary wall structure, with, on the one hand, very clear sublayers in the case of flax, date palm leaf sheath, and jute and, on the other hand, hardly distinguishable sublayers in the case of hemp and nettle with an irregular matrix like a grid for the cell corner middle lamella and sometimes a strong globular or nodular aspect for the primary wall in the case of kenaf. The global biochemical composition or the origin and the role of each kind of fiber in its respective plant does not completely explain the different kind of CCML encountered here, and local biochemical analysis will be necessary for the future. Nevertheless, the present results give complementary information for future modeling of plant fiber bundles and the design of better performing biocomposites. In addition to the semi-quantitative values provided, our results open new perspectives for future studies on the natural variability of the bundle intra plant (in the same stem all along its height) and inter plant (comparing similar locations in different stem samples) properties. 

## Figures and Tables

**Table 1:** Average and standard deviation of the indentation modulus obtained on the middle lamella of the panel of plant fibers considered.

Indentation Modulus (GPa)	
*Linum Usitatissimum L.* **(Flax)**	10.2 (± 1.2)
*Cannabis Sativa* **(Hemp)**	16.1 (± 1.4)
*Corchorus capsularis* **(Jute)**	10.9 (± 1.6)
*Hibiscus cannabinus* **(Kenaf)**	10.2 (± 2.2)
*Urtica Doïca* **(Nettle)**	14.7 (± 1.3)
*Phoenix dactylifera* **(Date palm leaf sheath)**	6.7 (± 0.9)

**Table 2:** Literature review of the biochemical global composition of the bundles of fibers studied, not reflecting the specific ML compositions.

	Cellulose (%)	Hemicellulose (%)	Lignin (%)	Reference
***Linum Usitatissimum L.* (Flax)**	60–85	14.0–20.6	1–3	[60,61,62]
***Cannabis Sativa*** **(Hemp)**	55–90	12	2–5	[63,64,65,66]
***Corchorus capsularis*** **(Jute)**	58.0–71.5	13.6–24.0	11.8–16	[67,68]
***Hibiscus cannabinus*** **(Kenaf)**	52.0–61.2	18.5–29.7	12.9–16.1	[69,70,71]
***Urtica Doïca*** **(Nettle)**	65.3–86.3	5.2–12.5	1.6–3.8	[72]
***Phoenix dactylifera*** **(Date palm leaf sheath)**	34–45.1	27.7–28.9	16.9–18.2	[51,73]
